# Neurophilic Descending Migration of Dorsal Midbrain Neurons Into the Hindbrain

**DOI:** 10.3389/fnana.2018.00096

**Published:** 2018-11-13

**Authors:** Claudia M. García-Peña, Daniela Ávila-González, Amaya Miquelajáuregui, Carlos Lozano-Flores, Grant S. Mastick, Elisa Tamariz, Alfredo Varela-Echavarría

**Affiliations:** ^1^Department of Developmental Neurobiology and Neurophysiology, Instituto de Neurobiología, Universidad Nacional Autónoma de México (UNAM), Querétaro, México; ^2^Department of Biology, University of Nevada, Reno, Reno, NV, United States

**Keywords:** embryo, migration, tyrosine hydroxylase, calbindin, neurophilic, midbrain, rat, mouse

## Abstract

Stereotypic cell migrations in the developing brain are fundamental for the proper patterning of brain regions and formation of neural networks. In this work, we uncovered in the developing rat, a population of neurons expressing tyrosine hydroxylase (TH) that migrates posteriorly from the alar plate of the midbrain, in neurophilic interaction with axons of the mesencephalic nucleus of the trigeminal nerve. A fraction of this population was also shown to traverse the mid-hindbrain boundary, reaching the vicinity of the locus coeruleus (LC) in rhombomere 1 (r1). This migratory population, however, does not have a noradrenergic (NA) phenotype and, in keeping with its midbrain origin, expresses Otx2 which is down regulated upon migration into the hindbrain. The interaction with the trigeminal mesencephalic axons is necessary for the arrangement and distribution of migratory cells as these aspects are dramatically altered in whole embryo cultures upon disruption of trigeminal axon projection by interfering with DCC function. Moreover, in mouse embryos in an equivalent developmental stage, we detected a cell population that also migrates caudally within the midbrain apposed to mesencephalic trigeminal axons but that does not express TH; a fraction of this population expresses calbindin instead. Overall, our work identified TH-expressing neurons from the rat midbrain alar plate that migrate tangentially over long distances within the midbrain and into the hindbrain by means of a close interaction with trigeminal mesencephalic axons. A different migratory population in this region and also in mouse embryos revealed diversity among the cells that follow this descending migratory pathway.

## Introduction

Early brain development is characterized by extensive neuronal migration, sometimes over long distances, following stereotypic routes and during precise time-windows. These migrations are crucial for the proper patterning of neural networks and their disruption has been implicated in numerous neuro-developmental disorders, such as schizophrenia (Lencz et al., 2013; Rakić et al., 2015), autism (Chuang et al., 2015), bipolar and depressive disorder (Goossens et al., 2003; Bertram et al., 2007; Li et al., 2011), mental disability and epilepsy (Reuter et al., 2014).

Based on the direction of displacement, neuronal migration has been defined as radial or tangential (Rakic, 1990; Marín and Rubenstein, 2003). Radial displacement occurs when cells from the ventricular germinal zone slide along the fibers of radial glia to reach the marginal zone and pial surface. This is also known as gliophilic migration and is important for the formation of layers in the developing cortex, hippocampus, cerebellum, diencephalon, brainstem and spinal cord (Rakic, 1971, 1990; de Carlos et al., 1996). Tangential migration is observed as neuroblasts move over the developing brain in routes parallel to the pial surface, as was demonstrated for cortical GABAergic interneurons and locus coeruleus (LC) noradrenergic (NA) neurons (Tanaka et al., 2006; Shi et al., 2008). Tangential migration can also occur along axons (“neurophilic” migration; Rakic, 1990; Nadarajah and Parnavelas, 2002; Ghashghaei et al., 2007) or move in chains following different substrates or signals from the brain microenvironment (Marín and Rubenstein, 2003). Tangential migration can take place over long distances and across regional borders in the brain, as has been shown for neurons moving from the diencephalon to the telencephalon, from the ganglionic eminences to the cortex, from the diencephalic-telencephalic boundary rostrally into the telencephalon, and from the hindbrain to the pontine tegmentum (Letinic and Rakic, 2001; Aroca et al., 2006; Shi et al., 2008; Miquelajáuregui et al., 2010).

In the anterior region of the brainstem, a diversity of neuronal types has been described that includes dopaminergic (DA; Simon et al., 2001; Chinta and Andersen, 2005; Prakash and Wurst, 2006), GABAergic, glutamatergic (Lahti et al., 2013), and NA neurons (Aroca et al., 2006). The phenotype decision of the neuroepithelial progenitors leading to each neuronal group depends upon the positional information in each of the morphogenetic territories and the concentration of signals that generate gradients in the anterior/posterior and in the dorsal/ventral axes. In the anterior brainstem, ventral expression of Shh and dorsal expression of Wnt1 intersect the region where Otx2 expression in the midbrain abuts Gbx2 expression in the rhombomere 1 (r1) of the hindbrain (Li and Joyner, 2001; Inoue et al., 2012). At this juncture, the isthmic organizer, or mid-hindbrain boundary region (MHB) is established, thus influencing the region’s morphogenesis via secretion of Wnt1, predominantly from the posterior boundary of the midbrain and FGF8 from the anterior edge of the hindbrain (Joyner et al., 2000). These signals are part of complex pathways such as those that lead to the differentiation of catecholaminergic neuron types; DA neurons in the midbrain and NA neurons in r1, both of which can be identified by expression of tyrosine hydroxylase (TH), a common enzyme in the biosynthetic routes of catecholamines (Prakash et al., 2006).

Migration of these neuronal types has been observed in the developing brainstem. Progenitors of some DA neurons migrate first radially and then tangentially (subpially) from the floor plate ventricular zone to the basal plate marginal zone forming the substantia nigra pars compacta, while some of these floor plate cells populate the ventral tegmental area (VTA) after a short radial migration (Yang et al., 2013; Bodea et al., 2014). These processes of DA precursor cell migration have been linked to the function of the netrin receptor DCC (Xu et al., 2010). At a later stage, GABAergic neurons populate the VTA using DA axons to reach their final location (Vasudevan et al., 2012). Other studies showed as well that NA neurons migrate ventrally from the ventral alar plate of r1 to their final location in the LC involving Netrin/DCC signaling (Shi et al., 2008).

In this work, we uncovered a population of neurons expressing TH that originates in the alar plate of the midbrain and migrates posteriorly along a longitudinal route in close apposition to axons of the trigeminal mesencephalic nucleus (TmesV) thus constituting a neurophilic migration. Some neurons from this population reach the anterior r1 region adjacent to the prospective LC in the hindbrain. In mouse embryos, similarly neurophilic migrating populations were detected that lack TH and one of them instead expresses calbindin, revealing heterogeneity among the descending migratory cells in this region.

## Materials and Methods

### Animals

Wistar rats were housed and used at Universidad Nacional Autónoma de México (UNAM) according to regulations of the Mexican government regarding the use of laboratory animals for research purposes (NOM-062-ZOO-1999). This protocol was approved by the Institute’s Research Ethics Commitee (Comité de Ética en la Investigación, INB-UNAM) with register #001. Animals were killed with CO_2_ and by cervical dislocation by trained personnel at various gestational days, using the morning of the day of detection of the vaginal plug as embryonic day (E) 0.5. For every experiment, 5–10 embryos for each condition were analyzed.

DCC mutant mice maintained at University of Nevada, Reno were a kind gift of Frederic Charron (ICMR, Montreal, CA, Canada). The mice were euthanized following NIH guidelines according to protocol #2015-00435. DCC genotyping was performed as previously described (Fazeli et al., 1997).

### Immunofluorescence

For whole brain immunostaining, embryos were fixed for 4 h in 3.5% paraformaldehyde (PFA) in phosphate buffer saline (PBS; 70011-044, Gibco, Life Technologies, Grand Island, NY, USA). Embryos were then washed 10 times per 10 min each in PBS and the brains were extracted. Brains were treated with 0.3% Triton X-100 in PBS for 30 min and then incubated for 30 min in 10% goat serum (16210072, Gibco) or horse serum (26050070, Gibco) in PBS. After five washes of 10 min each in PBS, brains were incubated at 4°C for 16 h with primary antibodies diluted in 1% Triton X-100 in PBS for membrane proteins or 3% Triton X-100 in PBS for transcription factors, both containing 5% horse or goat serum. Brains were then washed 10 times for 10 min each followed by an incubation with horse or goat serum in PBS as described above. Incubation for 1 h with secondary antibodies (1:1,000) diluted in PBS was followed by 10 washes for 10 min each in PBS. Brains were then hemisected by cutting along dorsal and ventral midlines and mounted flat in coverslips in 0.4 mg/ml DABCO 33 LV (290734, Sigma-Aldrich, St. Louis, MO, USA) in 10% glycerol (g9012, Sigma-Aldrich) in PBS with the pial side up. Boundaries between the different brain regions were defined based on the morphology of the flat-mounted specimens.

For immunostaining on brain sections, embryos were fixed in 3.5% PFA in PBS, washed as mentioned above, cryoprotected in 30% sucrose in PBS, and frozen in Tissue-Tek (Sakura Finetek, Torrance, CA, USA). Cryostat sections (20 μm) were then obtained and collected in Fisherbrand Superfrost Plus Microscope Slides (Fisher Scientific International, Pittsburgh, PE, USA). The sections were dried up for 4 h at room temperature and stored at −20°C until use. For immunostaining, sections were washed several times in PBS, incubated for 30 min in 5% goat or horse serum in PBS, and then washed three times for 5 min each with PBS. Incubation of primary antibodies was performed for 16 h in 1% Triton X-100 in PBS containing 5% horse or goat serum at 4°C. The sections were then washed with PBS three times for 5 min each. The sections were then incubated with secondary antibodies at room temperature for 1 h in PBS containing 5% horse or goat serum. After incubation, sections were washed with PBS five times for 5 min each and mounted with coverslips with DABCO/glycerol as described above.

The primary antibodies and registry IDs^1^ for the following protein targets and dilutions were: TH made in rabbit (1:1,000, AB_2617184, Pel-Freez, Rogers, AR, USA); TH made in sheep (1:150, AB_461070, Pel-Freez); calbindin D28K (1:500, AB_2068336, Millipore, Billerica, MA, USA); DBH made in mouse (1:500, AB_2245740, Millipore); Otx2 made in rabbit (1:500, AB_776930, Abcam, Cambridge, MA, USA); Phox2a made in mouse (1:400, AB_944807, Abcam); Phox2b made in rabbit (1:400, AB_10675986, Abcam); and β-III-Tubulin made in mouse (1:2,000, AB_2313773, Covance, Princeton, NJ, USA).

The secondary antibodies used were: Alexa-Fluor 488 goat anti-mouse (1:1,000, AB_138404, Invitrogen, Eugene, OR, USA), Alexa-Fluor 488 goat anti-rabbit (AB_2576217, Invitrogen), Alexa-Fluor 488 donkey anti-sheep (AB_141362, Invitrogen), Cy3 goat anti-mouse (AB_2340817, Jakson ImmunoResearch, West Grove, PA, USA) and Cy3 goat anti-rabbit (AB_955021, Abcam). For chromogenic immunostaining, peroxidase anti-rabbit (AB_90264, Millipore) secondary antibodies were used and revealed with diaminobencidine (DAB; D8001, Sigma-Aldrich) and hydrogen peroxide (216763, Sigma-Aldrich).

### Image Acquisition

**Figure 1:**
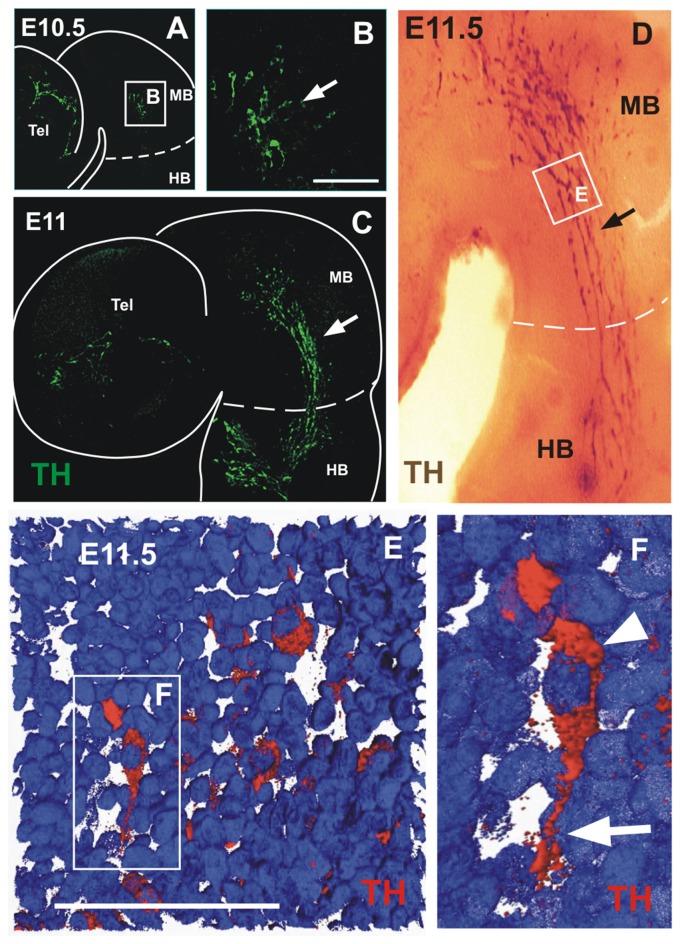
A novel population of tyrosine hydroxylase (TH)-expressing cells in the midbrain alar plate. Immunofluorescent staining for TH in flat-mounted E10.5–11.5 hemi-brains is shown in (**A–F)**. In all panels, rostral is to the left and dashed lines indicate the approximate location of the mid-hindbrain boundary region (MHB). Arrows in **(A,C,D)** indicate the location of the TH-expressing cell population that is present in the hindbrain from E10.5 to E11.5. **(B,E,F)** Magnified views of the areas indicated by frames in **(A,D,F)**, respectively. **(D)** TH chromogenic immunostaining (not flat-mounted) revealing the location of the midbrain TH^+^ cell population that is continuous into the hindbrain. **(E)** Volume reconstruction (3D) of a Z confocal series of a magnified region of the TH^+^ cell cluster showing a few stained cells (red) and cell nuclei stained with DAPI in blue. This image was obtained from a parasaggittal cryosection of approximate location as indicated in **(E,F)**. Single TH^+^ cell showing its leading process (arrow) toward the hindbrain as observed in most individually identified neurons revealing a precise pattern in this cell group; its cell body containing the nucleus is indicated by the arrowhead. MB, midbrain; HB, hindbrain; Tel, telencephalic vesicle. Scale bars: 100 μm.

**Figure 2:**
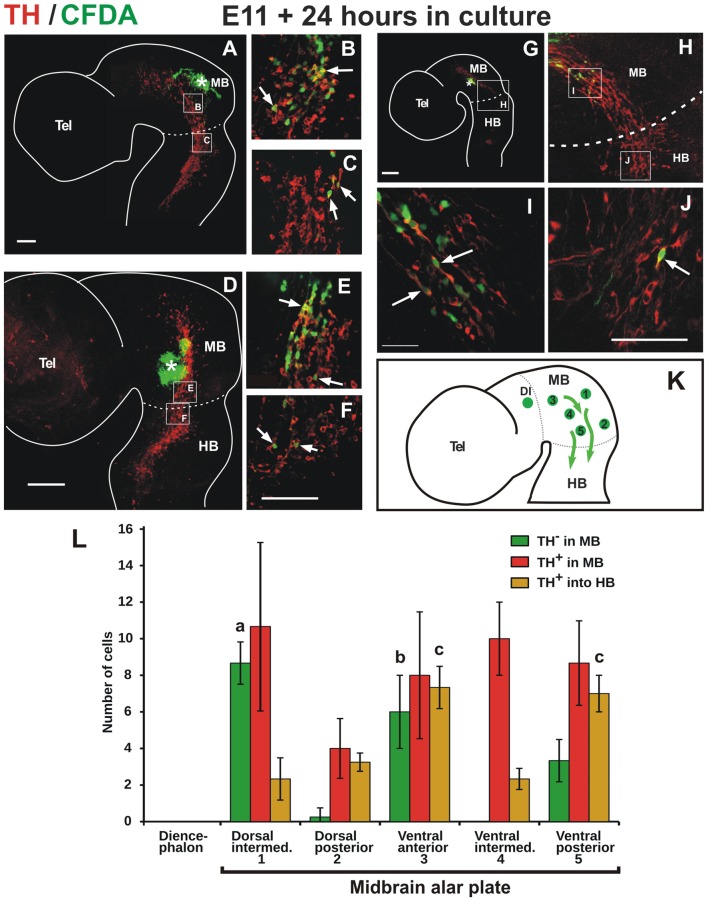
TH cells from the alar plate of the midbrain migrate descendingly and some reach the anterior end of the hindbrain. Flat-mounted hemibrains of embryos injected with carboxyl-fluorescein di-acetate (CFDA, green) at various positions of the midbrain (indicated with asterisks) and cultured for 24 h, followed by TH immunostaining (red). In all panels, rostral is to the left and dashed lines indicate the approximate location of the MHB. **(A–C)** Labeling from the dorsal aspect of the midbrain reveals CFDA^+^/TH^+^ cells that migrate ventrally into the TH^+^ cell cluster and in a posterior direction along the cluster. Labeling from the posterior end of the midbrain **(D,E)** and from an intermediate position along its antero-posterior extent **(G–J)**, reveal CFDA^+^/TH^+^ cells that migrate in a posterior direction along the ventral region of the alar plate. From all labeled positions shown, cells were observed to reach the anterior region of the hindbrain (arrows in **C,F,J**). **(B,C,E,F,I,J)** Magnifications of the regions indicated by frames in **(A,D,G,H)**. Location of **(H)** is indicated in **(G)**. **(K)** Summary diagram of various labeling points in the midbrain (green circles) and the direction of migration (arrows). Numbers in the diagram correspond to labeling regions in the midbrain alar plate indicated in **(L)**. The green circle in the diencephalon in **(K)** indicates labeling at the pretectum from which no migratory cells were observed (not shown). Panel **(L)** shows the quantitation of CFDA-labeled cells from various regions in the midbrain alar plate. Total migratory cells include all labeled cells that could be identified away from the labeling points; these include cells that were observed in the midbrain that do not express TH (TH^−^ in MB), cells in the midbrain that express TH (TH^+^ in MB), and cells that express TH that reached the anterior region of r1 in the hindbrain (TH^−^ in HB). Standard deviation is indicated in each of the bars. Value (a) indicates significant difference (*p* < 0.01) from 2, 4 and 5; (b) difference from 2 and 4 and (c) difference from 1, 2 and 4. Number of injections indicated in “Materials and Methods” section. Scale bars: 100 μm. Bar in **(F)** also applies to **(B,C,E)**; bar in **(J)** also applies to **(I)**.

**Figure 3:**
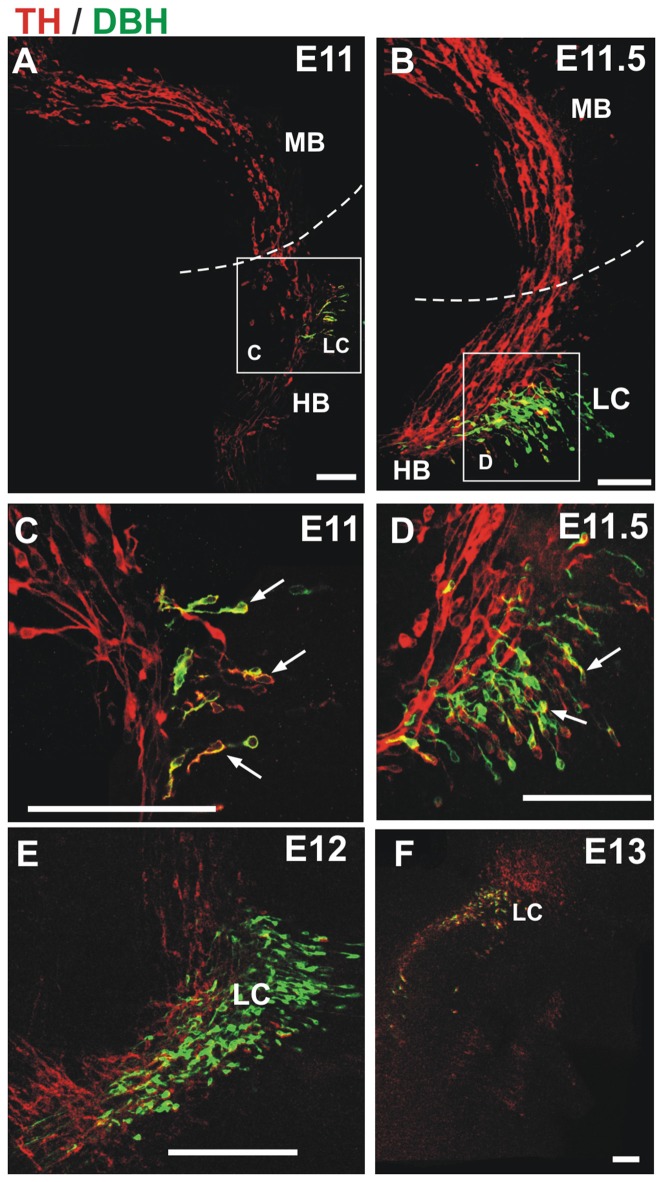
TH migratory cells from the midbrain do not express the noradrenergic (NA) marker dopamine β-hydroxylase (DBH). Double immunostaining for TH (red) and DBH (green) was performed in hemibrains. In all panels, rostral is to the left and dashed lines indicate the approximate location of the MHB. Migratory cells from the midbrain (TH-only) appear to merge with TH^+^/DBH^+^ cells (arrows) in the hindbrain at E11 and E11.5 **(A–D)**. **(C,D)** Magnified views of frames indicated in **(A,B)**, respectively; **(E,F)** by E12 and E13 two distinct populations (TH-only and TH/DBH double labeled cells) are detected at the rostral end of the hindbrain. Scale bars: 100 μm.

**Figure 4:**
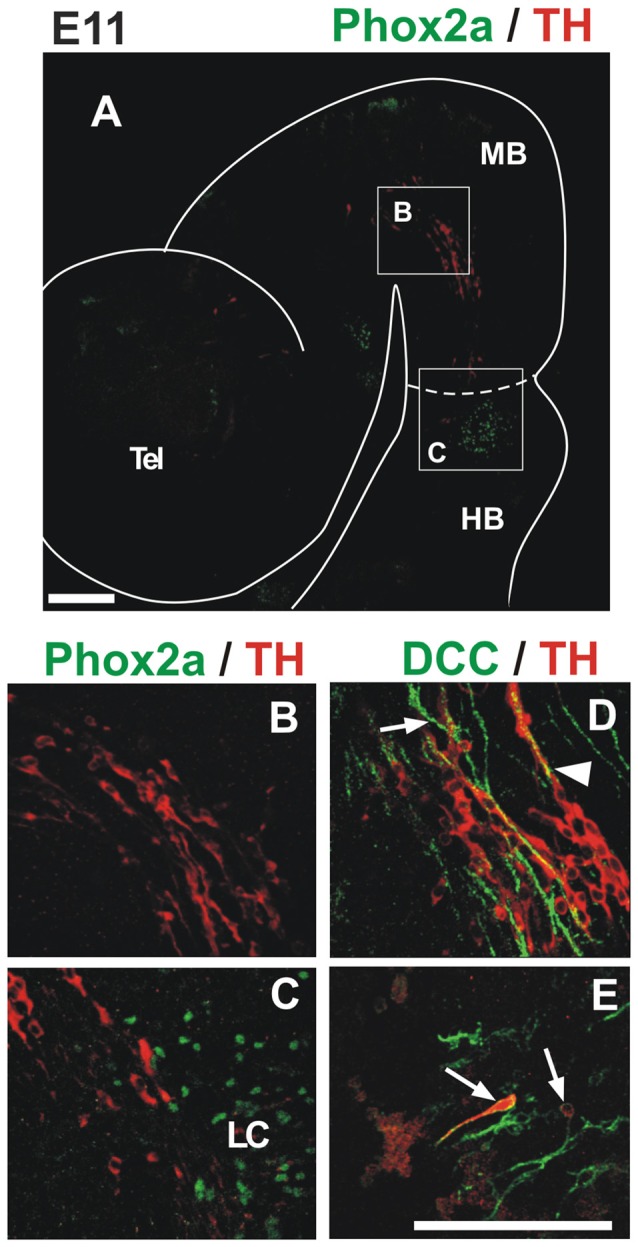
TH migratory cells from the midbrain do not express the markers Phox2a and DCC present in LC cells. Double immunostaining for TH (red) and either Phox2a **(A–C)** or DCC **(D,E)** reveals that TH^+^ migratory cells from the midbrain do not express these markers found in NA neurons at this stage. In all panels, rostral is to the left and dashed lines indicate the MHB. Note that although expression of DCC was absent from TH migratory cells **(D)**, it stained TH^+^ cells of the prospective LC (arrows in **(E)**) and longitudinal axons in the midbrain (arrow in **(D)**). Arrowhead in **(D)** indicates apposition of DCC axons and TH cells. Location of **(B,C)** is indicated in **(A)**. Panels **(D,E)** correspond to locations similar to **(B,C)**, respectively. LC, locus coeruleus. Scale bars: 100 μm. Bar in **(E)** also applies to **(B–D)**.

**Figure 5:**
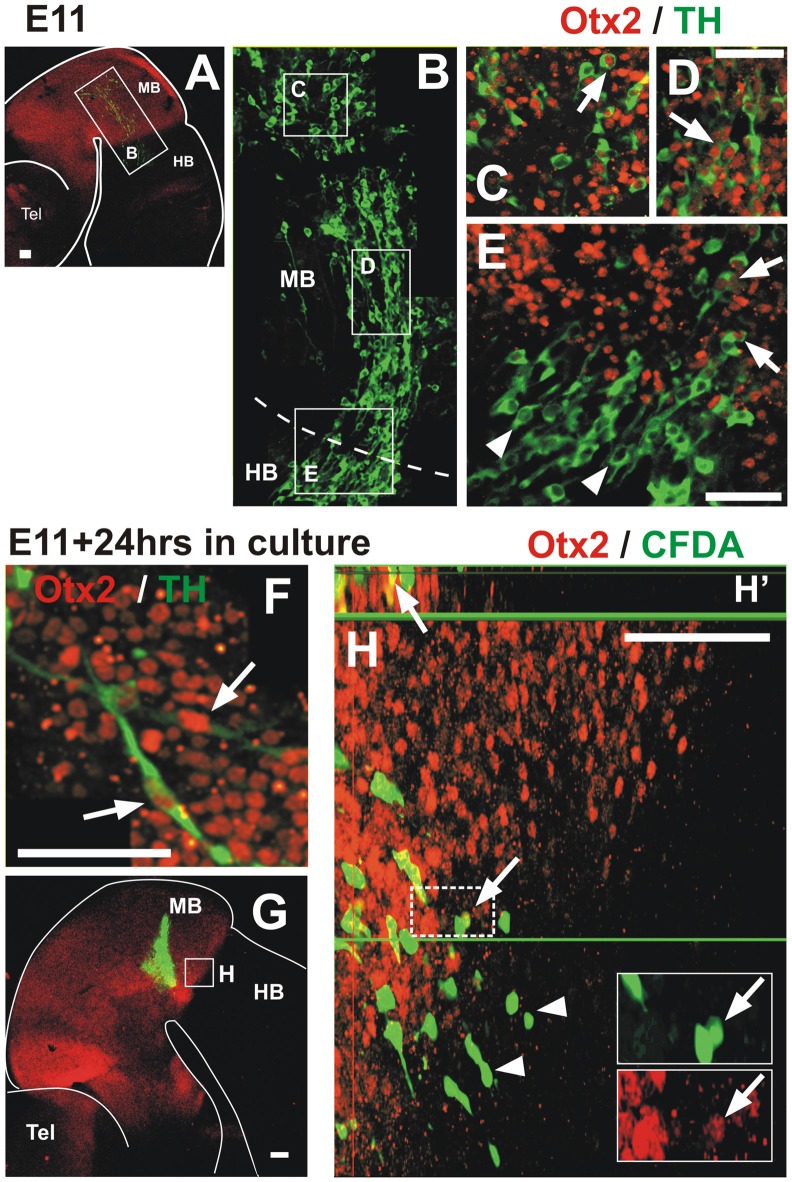
Migratory TH^+^ cells from the midbrain down-regulate Otx2 expression along their pathway into the hindbrain. In all panels, rostral is to the left and dashed lines indicate the MHB. **(A,B)** Double immunostaining of Otx2 (red) and TH (green) reveals co-expression in the midbrain; a mosaic reconstruction of individual micrographs of the whole extent of the TH cell cluster is shown in **(B)**, its location is indicated in **(A)**. **(C–E)**. Magnified views of the regions indicated in **(B)** showing Otx2 and TH co-expression (arrows). Panels **(C,D)** correspond to regions within the midbrain and **(E)** corresponds to the MHB region and shows lack of expression of Otx2 in TH cells in the hindbrain territory (arrowheads). Panel **(F)** corresponds to a location similar to **(D)** of an E11 rat embryo cultured for 24 h; it shows Otx2 and TH co-expression (arrows). **(G,H)** CFDA labeling (green) followed by culture and Otx2 immunostaining (red). **(H)** Magnified view of the region indicated in **(G)** showing CFDA labeled cells expressing Otx2 in the midbrain territory (arrows) and lack of expression in the hindbrain territory (arrowheads). Insets in **(H)** represent magnified views of the cells in the dashed white frame showing green and red channels; arrow indicates CFDA-labeled cell (green) that expresses Otx2 (red). Panel **(H′)** shows digital orthogonal projection of plane indicated by green horizontal line showing CFDA-labeled cell that expresses Otx2 (arrow). Scale bars: 100 μm. Bar in **(D)** also applies to **(C)**.

**Figure 6:**
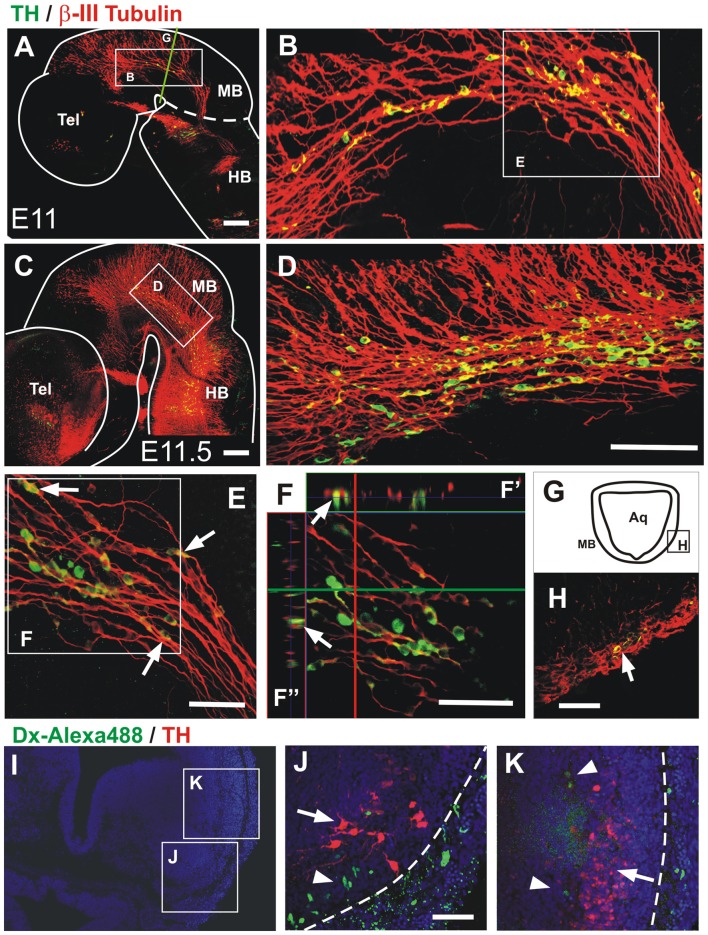
Midbrain TH^+^ neurons migrate in close apposition to axons of the mesencephalic trigeminal nucleus (TmesV). Double immunostaining for TH (green) and β-III tubulin (red) confirms the neuronal identity of TH-expressing cells in the midbrain alar plate and reveals their close apposition to TmesV axons along their migratory route at E11 **(A,B,E,F,H)** and E11.5 **(C,D)**. **(B,D)** Magnifications of the regions indicated by frames in **(A,C)**, respectively. Panel **(E)** is a higher magnification image of the region indicated in **(B)** and shows TH cells apposed to axons (arrows). **(F)** Single frame of a confocal Z-series from the image shown in **(E)**, indicating with the green horizontal line and the red vertical line the position of the digital sections on the orthogonal projection of the Z-series shown on **(F′,F″)**, respectively, and confirming the close apposition of TH neurons to TmesV axons. **(H)** Transverse section of the midbrain at E11 (its approximate location is indicated by frame in **(G)** and by the green line in **(A)**), showing double immunostaining for TH and β-III tubulin indicating that TH neurons are surrounded by axon bundles of the TmesV (arrow). **(I–K)** TmesV neurons were labeled from the trigeminal ganglion in rat embryos (E12.5 + 6 h) do not express TH. Embryos of E12.5 + 6 h were labeled from the trigeminal ganglion with Dextran Alexa Fluor 488 (green) followed by TH immunostaining (red). Panel **(I)** shows a panoramic view of a transverse section at the anterior end of r1 and the location of **(J)** and **(K)**. Arrow in **(J)** indicates the developing LC and arrowhead indicates labeled TmesV cells. Arrow in **(K)** indicates a lateral cluster of cells that is continuous with the TH expressing territory in the ventral rim of the alar plate in the midbrain and arrowheads indicate labeled TmesV cells. Dotted lines indicate the exterior boundary of the developing brainstem. Scale bars: **(A–H)** 100 μm. Bar in **(D)** also applies to **(B)**. **(J,K)** 50 μm.

**Figure 7:**
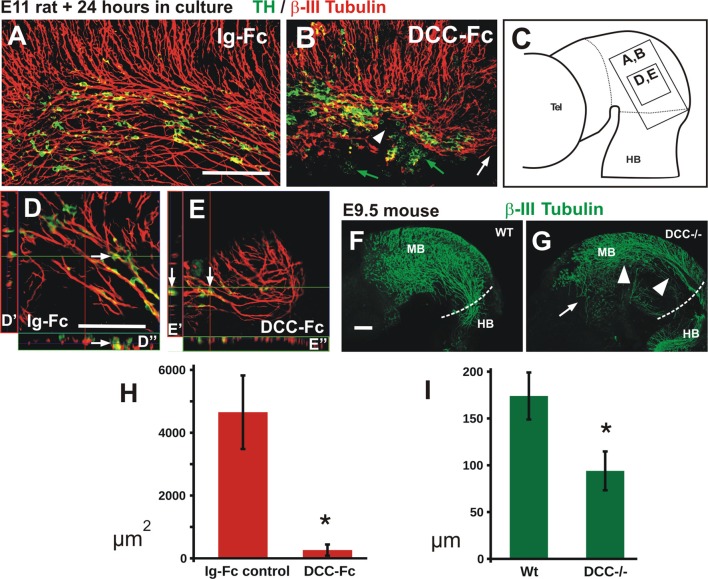
Blocking DCC signaling in rat embryos impairs TmesV axon projection and TH cell migration, and TmesV axons misproject in DCC mutant mouse embryos. Embryos cultured in presence of Ig-Fc as control **(A,D)** or with the DCC-Fc chimera to alter TmesV axon projection **(B,E)** followed by double immunostaining for TH and β-III tubulin; their approximate location is indicated in diagram in **(C)**. Overall organization of TmesV neurons and their axon projection was dramatically altered by DCC-Fc **(B,E)** along with a drastic disorganization of TH neurons and their impaired caudal migration into the hindbrain. White arrows in **(D,E)** indicate TH^+^ cells apposed to β-III tubulin axons in single optical sections and orthogonal digital projections (**D′,D″** and **E′,E″** respectively); in **(B)** green arrows indicate TH^+^ cells that do not interact with β-III tubulin axons, white arrowhead indicates interruption of the longitudinal tract of the TmesV, and white arrow indicates lack of projection of the TmesV into the hindbrain. Panel **(F)** is a flat-mounted hemibrain of a wild-type mouse embryo stained for β-III tubulin and **(G)** is from a DCC^−/−^ embryo. Arrow in **(G)** indicates misprojecting axons and arrowheads indicate dorsal displacement of the TmesV axon bundle. **(H)** The treatment with DCC-Fc reduced the density of TmesV axon bundles. The area covered by β-III tubulin axon bundles in projections of confocal Z-stacks was measured. Asterisk indicates statistical difference between controls and DCC-Fc treated embryos (Wilcoxon-Mann-Whitney test, *p* = 0.0143). **(I)** The TmesV axon bundle was dorsally displaced in DCC^−/−^ embryos. The distance of the center of the axon bundle from the dorsal edge of the brain was measured at the isthmic region for wild-type and mutant embryos. Dorsal displacement is revealed by a significantly shorter distance of the bundle in mutant embryos. Asterisk indicates statistical difference between controls and DCC^−/−^ embryos (Wilcoxon-Mann-Whitney test, *p* = 0.008). Scale bar: 100 μm. Bars in **(A,D,F)** also apply to **(B,E,G)**, respectively.

**Figure 8:**
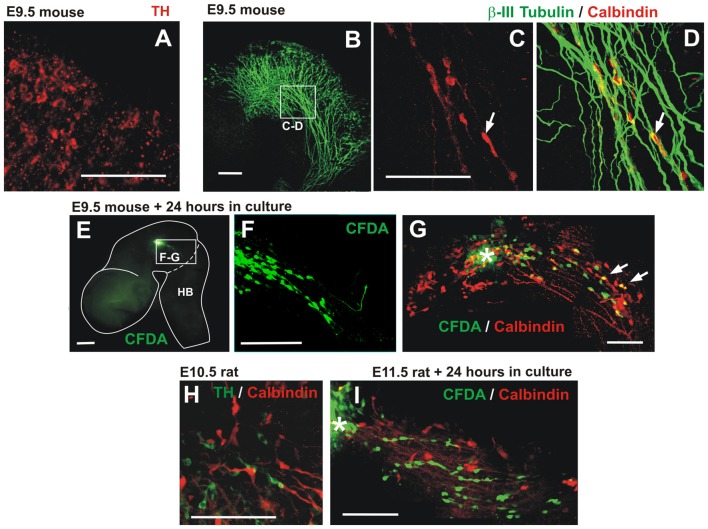
Descending migratory population of calbindin-expressing cells in mouse embryos. **(A)** E9.5 mouse embryonic midbrain immunostained for TH showing a low-expressing population in the dorsal-most aspect of the midbrain near its boundary with the pretectum. **(B–D)** E9.5 mouse midbrain showing calbindin cells apposed to TmesV axons (β-III tubulin). Panels **(C,D)** are red and merged red/green channels, respectively, of the same magnified region indicated in **(B)**. **(E,F)** CFDA labeling was performed in the midbrain of E9.5 embryos followed by 24 h in culture revealing caudally-migrating cells. Panel **(F)** is a magnification of the frame indicated in **(E)**. **(G)** Following CFDA labeling (asterisk) and culture, the midbrain was immunostained for calbindin showing that some migratory cells express this marker (arrows). **(H)** E10.5 rat embryonic midbrain immunostained for TH and calbindin in a location similar to **(C–D)**. **(I)** CFDA labeling in the midbrain of E11.5 rat embryos followed by 24 h in culture and immunostaining showing caudally-migrating cells that do not express calbindin. Scale bars: 100 μm.

Three different confocal microscopes were used: Nikon Eclipse E600 PCM 2000 (Figures 1A–C, 2, 6), Zeiss LSM510 META (Figures 1E,F, Figures 3–6, 7A,B,D,E, 8A,C,D,F–I). Figures 7F,G were taken with the Leica TCS confocal microscope. Unless otherwise indicated, for each image Z-stacks of pictures 1.5 μm apart were taken with a total of 20–30 pictures per sample; some stacks were then projected to a single image to show the entire signal present in the tissue. For orthogonal pictures, Z-stacks were digitally rotated 90°C and cut digitally in different areas of the sample to show co-localization of fluorescent labels in a single frame. Volume reconstruction (3D) of confocal Z-series was performed with ImageJ^2^ followed by Amira 6.4.0 (Thermo Fisher Scientific) using its isosurface function.

### Cell Lineage Labeling and Quantitation

To trace the migration of cells from their place of origin, we injected in whole embryos the lipophilic dye carboxyl-fluorescein di-acetate (CFDA, SE, 557 M.W., V12883, Invitrogen; 10 mM diluted in DMSO 472301, Sigma-Aldrich) which only fluoresces upon cell entry (García-Moreno et al., 2010). Injections were made in rat embryos at E11.5 and in mouse embryos at 9.5 in different areas of the ventricular surface of the diencephalon or midbrain, followed by incubation for 24 h in blood serum obtained from Wistar rats with a constant flow of a gas mix of 95% O_2_ and 5% CO_2_. After incubation, embryos were fixed with 3.5% PFA in PBS for 4 h and immunostaining was performed as described above.

To quantitate the migratory cells from various regions of the midbrain, ventricular injections were applied at the locations indicated in Figure 2K followed by TH immunostaining and flat-mounting. For each injection, Z-stacks of confocal images were obtained from regions containing all CFDA labeled cells that migrated away from the labeling point among which TH^+^ and TH^−^ cells were counted. Results were analyzed with the program JASP 0.8.6.0 and JASB 0.9.0.1 applying an ANOVA with *post hoc* Tukey test. The number of injections analyzed for each region indicated in Figure 2 were: **DI**, 2; **1**, 3; **2**, 4; **3**, 3; **4**, 3; and **5**, 3.

### Retrograde Labeling With a Fluorescent Dye

For the retrograde labeling we employed a variant of a method described previously (Auclair et al., 1999). Rat embryos were extracted from the pregnant rat female and placed in ice-cold DMEM/F12 (51445C, SIGMA). Embryos were immobilized with minutien pins on a 60 mm petri dish with a bed of silicon gel submerged in the same medium with one of their sides facing up. The ectoderm and mesenchyme were removed to expose the trigeminal ganglion which was then severed with the tip of a minutien pin laden with Dextran Alexa Fluor 488, 10,000 MW (D-22910; Molecular Probes, Invitrogen). After 3 min, the petri dish was immersed in 250 ml of medium at 25°C, bubbled with a gas mixture of 95% O_2_ and 5% CO_2_ for 3 h, and fixed with 3.5% PFA and immunostained as described above.

### Blocking DCC-Mediated Signaling in Cultured Embryos

To block the function of DCC in cultured embryos, we added the DCC-Fc chimeric protein (20 ng/ml, 844-DC-050, R&D Systems, Minneapolis, MN, USA) to the incubation medium of whole embryos in culture as described elsewhere (García-Peña et al., 2014). After incubation for 24 h, embryos were fixed, whole brains were immunostained, and hemibrains mounted flat as described above. For control experiments, Recombinant Human IgG1 Fc (110-Hg-1-100, R&D Systems) was used at 20 ng/ml as in previous studies (Liu et al., 2011; Czajkowsky et al., 2012; García-Peña et al., 2014). To quantitate the effect of DCC-Fc on the TmesV axon bundles, four Z-stacks per experimental condition (36 confocal planes, 0.7 μm apart) were obtained of a microscope field of 225 × 225 μm (4.551 pixels/μm) of the intermediate ventral region on the midbrain alar plate, images were projected onto a single plane, the automatic Yen threshold method of ImageJ^3^ was used to convert all images from grayscale to black and white and the total area occupied by the axon bundles was measured for each image. The Wilcoxon-Mann-Whitney test calculator (Marx et al., 2016) was employed to assess statistical differences between controls and Fc-DCC treated cultures.

### Analysis of DCC Mutant Embryos

For the analysis of TmesV axon distribution in control and DCC mutant embryos, brains were extracted, immunostained for β-III-Tubulin (1:2,000, AB_2313773, Covance), and hemibrains were mounted flat. The distance from the center of the TmesV axon bundle to the dorsal edge of the brain was measured at the isthmic region for five wild-type and five mutant embryos. The Wilcoxon-Mann-Whitney test calculator (Marx et al., 2016) was employed to assess statistical differences between controls and mutant embryos.

## Results

### TH-Expressing Cells Migrate From the Midbrain to the Hindbrain in Rat Embryos

Analyzing cells expressing TH in the developing rat midbrain, we detected a population that precedes the appearance of the widely studied ventral DA neurons of the substantia nigra and VTA. At E10.5, a few scattered TH^+^ cells appear in the alar plate of the midbrain and in the telencephalon (Figures 1A,B). After 12 h, and remaining up to E11.5, cells in the midbrain increase in number and display a distinctive elongated morphology and a clustered cell arrangement stretching from the anterior end of the midbrain to the anterior end of the hindbrain (Figures 1C,D). This cell distribution and the caudal orientation of their elongated cell processes (Figures 1E,F), suggested a migratory stream along the ventral border of the alar region that extended to the hindbrain region containing the prospective LC (Shi et al., 2008). Half a day later, at E12, the TH^+^ cells were no longer detected in the alar plate of the midbrain but a distinct cluster remains in the alar plate of r1 (not shown). Hence, these TH cells are an early-born population emanating from the alar aspect of the rat midbrain that seems to migrate to the hindbrain by E11.5.

To test directly the idea that this midbrain TH^+^ lineage is indeed migratory, and to characterize its possible displacement pathway, we performed labeling in several regions of the ventricular germinal zone of E11 embryos using the fluorescent cell tracer CFDA. After 24 h in culture, we found that the labeled midbrain progenitors gave rise to TH-expressing cells that migrated tangentially in a posterior direction from the injection sites (Figure 2). Labeling along the longitudinal extent of the TH^+^ domain revealed the caudal migration (Figures 2D–J) and even the dorsal-most midbrain region showed a contribution to this migratory population (Figures 2A–C). Strikingly, from most labeling points cells were observed to descend hundreds of micrometers into the anterior region of r1 adjacent to the prospective LC (Figures 2C,F,J).

Quantitation of the CFDA-labeled cells from the different labeling points revealed that TH cells that migrate within the midbrain and cells that reach the hindbrain arise from all labeled regions, and that the latter are generated in larger numbers from regions in the ventral alar plate (Figures 2K,L). Moreover, we observed that the dorsal intermediate and ventral anterior regions gave rise to the largest number of labeled cells that lack TH expression. In contrast, CFDA-injections in the pretectum and in the anterior and dorsal end of the midbrain revealed that no migratory cells emanate from these regions (not shown, DI indicated in Figures 2K,L).

Hence, these studies revealed a population of TH-positive cells that descends migrating (dm-TH) following a tangential pathway along the ventral region of the alar plate of the midbrain and traverse the MHB into the hindbrain (summary diagram in Figure 2K). It is important to note the detection of the additional population lacking TH that migrates along a similar descending route within the midbrain which did not reveal migration into the hindbrain (Figure 2L).

### Midbrain Descending Migratory TH Cells Converge at a Region Adjacent to the Prospective Locus Coeruleus in the Hindbrain

Since we observed that dm-TH cells migrated to r1 in the vicinity of the developing LC, we performed immunostaining for a marker for the latter, namely, the NA marker DBH, together with the reference staining for TH (Figure 3). From E11 to E12, this staining revealed the TH^+^/DBH^−^ dm-TH cells in midbrain and isthmic regions and TH^+^/DBH^+^ cells of the prospective LC at the anterior region of r1. At E11 and E11.5, TH^+^/DBH^+^ neurons were found in r1 as previously described (Aroca et al., 2006) and perpendicular to the dm-TH (Figures 3A–D). Later, at E12, a discrete TH^+^/DBH^−^ population was detected in the hindbrain, adjacent to LC TH^+^/DBH^+^ neurons which were also present at E13 (Figures 3E,F).

To characterize further the migratory cells, we performed double staining for TH and Phox2a or DCC (Figure 4), two additional markers that have been shown to label NA LC neurons (Morin et al., 1997; Shi et al., 2008). DCC label was detected in longitudinal axons of the midbrain (Figure 4D, arrow) but no labeling of dm-TH cells by DCC or Phox2a was observed in this region. Some TH cells, however, were apposed to the DCC axons which, based on their location, appear to correspond to the fibers of the TmesV (Figure 4D, arrowhead; Easter et al., 1993; Mastick and Easter, 1996). Additionally, as expected by the previous evidence, both markers were found in the region containing the developing LC (Figures 4A,C,E). Hence, these results show that dm-TH cells do not express NA markers during their migration within de midbrain or into the anterior region of r1.

To determine whether the dm-TH cells retain expression of markers of their region of origin as they migrate into the hindbrain, we performed double immunostaining for TH and the transcription factor Otx2 which is expressed in the midbrain and not in the hindbrain (Figures 5A–E). We observed that Otx2 is restricted to the forebrain and midbrain at E11 with a clear caudal limit of expression at the MHB (Figure 5A) as expected from previous studies (Li and Joyner, 2001; Inoue et al., 2012). We observed that dm-TH cells in the midbrain co-express Otx2 (Figures 5C–E, arrows) and that this expression decreases gradually as these cells get closer to the MHB, being completely absent in the hindbrain (Figure 5E, arrowheads). To confirm this down-regulation of Otx2 in migratory cells, we performed CFDA labeling in cultured embryos followed by Otx2 immunostaining. We confirmed that Otx2 is expressed in TH cells in the midbrain of unlabeled cultured embryos (Figure 5F, arrows) and that this expression decreases in CFDA labeled cells as they approach the MHB to disappear once the cells cross into the hindbrain (Figures 5G,H).

These findings indicate that dm-TH cells express Otx2 at their place of origin in the midbrain but lose this expression as they migrate into the hindbrain.

### Descending Migratory TH Neurons Navigate in Close Apposition to TmesV Axons

Since we found dm-TH cells apposed to longitudinal fibers expressing DCC in the midbrain (Figure 4D), we asked whether there was a neurophilic or axonophilic interaction of TH^+^ cells with longitudinal axon fibers. To answer this, we double-stained E11–11.5 brains for TH and the axon marker β-III tubulin. We observed that TH^+^ cells express β-III tubulin, thus revealing their neuronal identity (Moody et al., 1989), and that they were juxtaposed to the β-III tubulin-only axons of the TmesV (Figure 6; Mastick and Easter, 1996). Higher magnification views show that all TH^+^ cells detected were in close apposition to axon bundles along the whole longitudinal extent of the dm-TH stream (Figures 6B,D–F). The apposition of TH neurons to axon bundles was confirmed by orthogonal digital sections (Figures 6F,F’,F”) and in histological sections immunostained for TH and β-III tubulin (Figures 6G,H).

To assess further the proximity of the ventral alar plate TH domain to the TmesV, we performed retrograde labeling from the trigeminal ganglion in rat embryos with Dextran-Alexa488 and immunostaining for TH (Figures 6I–K). This allowed the labeling of the earliest projecting TmesV neurons at the anterior region of r1 at E12.5 plus 6 h (green, Figures 6J,K, arrowheads). Immunostaining with TH antibodies (red) revealed a population which by its ventrolateral location seems to correspond to the prospective LC (Figure 6J, arrow) and a faintly stained dorsolateral population (Figure 6K, arrow) at the ventral region of the alar plate. Retrogradely labeled TmesV neurons were found adjacent to both TH cell groups; external to the ventral group and internal to the dorsal group. Hence, these results confirm the proximity of the dorsal TH cell cluster to TmesV neurons which were found to lack TH expression.

As DCC is expressed by TmesV axons (Figure 4D), we hypothesized that this Netrin1 receptor has a role in TmesV axon guidance, and indirectly, in dm-TH migration. To test this idea, we interfered with DCC function in cultured E11 embryos with the aim of disrupting TmesV axon projection and assessing its effects in the dm-TH cells. For this we used a chimeric protein containing the DCC ectodomain fused to the immunoglobulin Fc domain (DCC-Fc) in cultured embryos. Control cultures with and without Ig-Fc molecules stained for TH and β-III tubulin, revealed normal distribution of TmesV axons and dm-TH neurons apposed to them as in non-cultured wild-type E11.5 embryos (Figures 7A,C,D,D′,D″). In contrast, in all nine embryos cultured with DCC-Fc (Figures 7B,C), the normal appearance of TmesV axons was drastically altered; the longitudinal axon bundles were greatly reduced, disorganized and discontinuous (arrowhead), and in most cultures failed altogether to reach the hindbrain (white arrow). Quantitation of the overall axon bundle density in the ventral alar plate region containing the TH cell population revealed a drastic reduction in Fc-DCC treated embryos compared to control clutures (Figure 7H). Concomitantly, the normal longitudinal alignment and distribution of dm-TH neurons was dramatically affected, and irregular clusters of these cells were observed (Figure 7B). It is important to note that most TH neurons remained closely associated to TmesV axons (9 embryos, Figure 7B and Figures 7E,E′,E″) while some TH neurons dissociated from axons in 3 out of 9 embryos (Figure 7B, green arrows). Overall, our findings suggest that dm-TH neurons depend on TmesV axons for their arrangement and descending migration.

Seeking confirmation of a role of DCC on TmesV axon projection, we analyzed wild-type and DCC knockout mouse embryos. We observed that by E9.5, axons in five wild-type embryos have reached the hindbrain, displaying a similar distribution to that observed in E11 rat embryos (Figure 7F), in agreement with the known developmental time difference between these two rodent species. In all five DCC^−/−^ embryos analyzed, however, axons in the anterior midbrain misprojected rostrally and ventrally (arrow, Figure 7G) and throughout its longitudinal extent, the TmesV axon bundle was dorsally displaced (arrowheads, Figures 7G,I). Hence, these results confirm a role for DCC in TmesV axon projection.

### Descending Migratory Cells Expressing Calbindin in the Embryonic Mouse Midbrain

In an attempt to identify the dm-TH in mouse embryos, we performed TH immunstaining as described for rat embryos. Analysis of E9–E10.5 mouse embryos, allowing for the developmental time difference between rat and mouse, revealed only at E9.5 a transient low TH expression at the dorsal-most aspect of the midbrain near its boundary with the pretectum (Figure 8A). A migratory TH population equivalent to that found in rat embryos, however, was not detected. In contrast, a small population of cells expressing the neuronal marker calbindin was found in the E9.5 mouse embryonic midbrain displaying a similar longitudinal distribution as dm-TH cells that was also apposed to TmesV axons (Figures 8B–D). Moreover, CFDA labeling in the midbrain of E9.5 mouse embryos followed by culture, revealed posteriorly migrating cells within the midbrain, some of which were calbindin-positive (Figures 8E–G). A similar calbindin population was found intermingled with TH cells in the midbrain of E10.5 rat embryos but these cells were not detected among the cells that migrate within the midbrain or into the hindbrain in cultured embryos upon CFDA labeling (Figures 8H,I and data not shown).

These findings confirm the existence of descending migratory cells within the mouse midbrain that also interact with TmesV axons but that differ in identity from those observed in rat embryos at equivalent stages.

## Discussion

In this study, we have uncovered a novel population of neurons expressing TH that migrates from the alar plate of the midbrain to the anterior region of the hindbrain in E10.5 to E11.5 rat embryos.

Labeling of these neurons with the fluorescent tracer CFDA in cultured embryos, revealed their origin at the whole longitudinal and dorsoventral extent of the midbrain alar plate region that also gives rise to the neurons of the TmesV (Mastick and Easter, 1996).

We also observed that the migratory cells converge into the alar plate ventral rim, in close apposition to the β-III tubulin-positive bundles of the descending TmesV axons (Mastick and Easter, 1996). Some neurons from this population (dm-TH), were seen to reach the anterior end of r1 in the hindbrain, in the vicinity of NA neurons of the prospective LC (Aroca et al., 2006). Their distinct midbrain origin was confirmed by labeling with ventricular injections in this territory, by their early expression of Otx2, and their lack of the markers Phox2a, DBH or DCC, expressed by LC neurons (Morin et al., 1997; Shi et al., 2008). Near the MHB, however, dm-TH neurons were observed to down-regulate Otx2 expression, presumably under the influence of this organizer region. These cells contrast with other migratory populations that have been identified following the markers of their regions of origin to apparently ectopic locations; a recent study uncovered one such population that migrates from the hypothalamus to the amygdala and retains in its final settling region the expression of Otp, a marker of their region of origin (García-Moreno et al., 2010).

The close interaction of dm-TH neurons with TmesV axons was revealed to be of relevance for the migratory population observed. Disrupting TmesV axon projection with a soluble version of the DCC ectodomain in cultured embryos, caused the disorganization of the dm-TH and prevented their clustering in the stream that reaches the hindbrain, thus indicating that TmesV axons are used by dm-TH neurons as neurophilic substrate during their descending displacement. Despite the dramatic alteration on the pathfinding of TmesV axons, however, most dm-TH neurons remained attached to them. This reveals a strong interaction, which is perhaps an important determinant of dm-TH migration. Incidentally, our studies also uncovered a role for DCC-mediated signaling in the TmesV axon pathfinding in rat embryos, also confirmed in mouse embryos lacking DCC. Hence, our results suggest that the role of DCC in dm-TH cell migration is mostly indirect by determining the TmesV axon projection. This contrasts with the role proposed for DCC in other developing neural cell types such as DA neurons (Xu et al., 2010), oligodendrocyte precursor cells (Spassky et al., 2002), dorsal spinal interneurons (Junge et al., 2016), and cerebellar neurons (Alcántara et al., 2000) in which it appears to mediate the direct effects of the chemotropic molecule netrin1.

In our studies, we also observed in the mouse embryonic midbrain, descending migratory cells in apposition to TmesV axons. These cells, however, differed from those observed in rat embryos in their lack of TH and in that a fraction of the mouse migratory cells express calbindin, thus revealing diversity among the migrating cell populations. Calbindin-expressing cells were also detected in the rat midbrain but no migration of these cells was observed.

The dorsal midbrain in mouse embryos has been previously described to contain transiently the TH-expressing A11 cell group first observed at E12.5 (Marín et al., 2005). Since this roughly corresponds to a stage in rat embryos (E13.5) which is at least two gestational days after the rat dm-TH population is last detected (E11.5), the equivalence between these two populations is unlikely and remains to be ascertained. It is also noteworthy that a similar population of TH-immunoreactive cells is present in the dorsal midbrain in human embryos (Puelles and Verney, 1998). Moreover, an earlier study employing a TH-lacZ transgene in mice, also described the presence of TH cells in the dorsolateral mesencephalon peaking from E9.5 to E10.5 (Son et al., 1996). An extensive search with our immunodetection approach, however, only detected a transient low TH-expressing population in the dorsal E9.5 midbrain and a search of TH mRNA by *in situ* hybridization at those stages did not reveal TH cells (Marín et al., 2005). This suggests that their TH expression levels are low and were better detected by the signal amplification afforded by ß-galactosidase in transgenic mice (Son et al., 1996). In another study in mouse embryos, labeling at E8.5 by a Wnt-1 expression-dependent reporter system, revealed a small number of cells that migrate from the midbrain into the ventral hindbrain, some of which express the serotoninergic marker 5-HT (Zervas et al., 2004). Hence, the differences in the timing of appearance of TH cells in the dorsal midbrain in the various species described and the differences we observed between the rat and mouse migratory cells reveal a population complexity that requires further comparative studies.

The crossing by neurons of boundaries between distinct morphogenetic brain territories is a source of neuronal diversity involved in the completion of neuronal repertoires in diverse brain regions (García-Moreno et al., 2010; Rudolph et al., 2010). Cortical GABAergic neurons, for example, originate in the ganglionic eminences and migrate to populate the developing cortex (de Carlos et al., 1996) passing across boundaries of Wnt signals and thus contributing interneurons to the cortical plate (Quinlan et al., 2009). Similarly, the dm-TH contributes TH^+^/DBH^−^ neurons to a cell cluster adjacent to the LC. Since our studies are limited to a narrow time window in the developing brain, further studies are necessary to determine whether the midbrain migratory neurons remain adjacent to the adult LC, as well as their fate and functional implications.

Our findings reveal a TH cell group in the dorsal midbrain of rat embryos that migrates posteriorly, with some cells reaching the vicinity of the LC well within the hindbrain. It is unlikely that these neurons are catecholaminergic or belong to the LC, however, owing to their midbrain origin and their lack of expression of Phox2a, DBH and DCC. On the other hand, the origin of these cells in the alar plate of the midbrain, their close association to TmesV axons during their migration, and the proximity to the LC of those that reach the hindbrain, suggest that these neurons are a lineage related to TmesV neurons, although their phenotypes are clearly different. The idea of the migration of midbrain TmesV neurons into the hindbrain has been previously suggested (reviewed in Lazarov, 2002). The possible projection of dm-TH neurons along the TmesV axon pathway, however, could not be confirmed in this study as our cell migration assay is limited to a short developmental time window and no expression of the TmesV markers E1.9 and B30 was detected at these early stages (not shown; Stainier and Gilbert, 1989, 1990). Moreover, we observed that the earliest projecting TmesV axons do not appear to express TH.

Hence, we describe in rat embryos a novel TH neuronal population that follows a hitherto unknown neurophilic or axonophilic migratory pathway apposed to TmesV axons within the midbrain alar plate and into the anterior region of r1 in the hindbrain adjacent to the prospective LC. This population, which does not share origin or identity with LC cells, seems to be related in lineage to TmesV neurons although their phenotypes are clearly different. An additional population which does not express TH was also detected in parallel to originate in the midbrain alar plate and to migrate caudally as well but with no evidence of migration into the hindbrain. These cell populations, along with similar descending cells but with different phenotypes in the mouse embryonic midbrain, warrant further studies to ascertain their fate, functional relevance and interspecies divergence.

## Author Contributions

CG-P, ET and AV-E conceived the project and designed the experiments. CG-P, DÁ-G, AM and CL-F performed the experiments and analyzed the data. GM designed the experiments and interpreted results. CG-P, AM and AV-E wrote the article. AV-E and ET supervised the project.

## Conflict of Interest Statement

The authors declare that the research was conducted in the absence of any commercial or financial relationships that could be construed as a potential conflict of interest.

## Footnotes

^1^http://antibodyregistry.org

^2^https://imagej.nih.gov

^3^https://imagej.nih.gov
